# Baseline-Free Structural Damage Identification for Beam-Like Structures Using Curvature Waveforms of Propagating Flexural Waves

**DOI:** 10.3390/s21072453

**Published:** 2021-04-02

**Authors:** Y. F. Xu, J. S. Kim

**Affiliations:** Department of Mechanical and Materials Engineering, University of Cincinnati, Cincinnati, OH 45221, USA; kim5jk@mail.uc.edu

**Keywords:** baseline-free structural damage identification, beam-like structure, curvature waveform, non-destructive evaluation, damage index

## Abstract

Curvatures in mode shapes and operating deflection shapes have been extensively studied for vibration-based structural damage identification in recent decades. Curvatures of mode shapes and operating deflection shapes have proved capable of localizing and manifesting local effects of damage on mode shapes and operating deflection shapes in forms of local anomalies. The damage can be inversely identified in the neighborhoods of the anomalies that exist in the curvatures. Meanwhile, propagating flexural waves have also been extensively studied for structural damage identification and proved to be effective, thanks to their high damage-sensitivity and long range of propagation. In this work, a baseline-free structural damage identification method is developed for beam-like structures using curvature waveforms of propagating flexural waves. A multi-resolution local-regression temporal-spatial curvature damage index (TSCDI) is defined in a pointwise manner. A two-dimensional auxiliary TSCDI and a one-dimensional auxiliary damage index are developed to further assist the identification. Two major advantages of the proposed method are: (1) curvature waveforms of propagating flexural waves have relatively high signal-to-noise ratios due to the use of a multi-resolution central finite difference scheme, so that the local effects of the damage can be manifested, and (2) the proposed method does not require quantitative knowledge of a pristine structure associated with a structure to be examined, such as its material properties, waveforms of propagating flexural waves and boundary conditions. Numerical and experimental investigations of the proposed method are conducted on damaged beam-like structures, and the effectiveness of the proposed method is verified by the results of the investigations.

## 1. Introduction

Vibration-based damage identification has been a major research topic of structural dynamics applications in recent decades. When local damage occurs to a structure, its local stiffness and/or mass will be quantitatively changed [1,2,3,4]. As a result, vibration characteristics of the structure, such as natural frequencies, mode shapes and operating deflection shapes, will be quantitatively changed. The changes in natural frequencies are considered global, as they can be estimated with a few measurements of frequency response functions of the structure, which can correspond to excitation and response points away from the damage [5,6]. The changes in mode shape and operating deflection shapes are considered local. The reason for this is that effects of the damage on the mode shapes and operating deflection shapes can be reflected when the locality of the damage falls within a measurement grid of the mode shapes and operating deflection shapes. Otherwise, the damage cannot be identified and such identification results are considered false-positives. Hence, wide and dense measurement grids are usually assigned onto the structure.

However, damage identification based on changes in mode shapes and operating deflection shapes is considered more robust than that based on changes in natural frequencies, for two reasons: (1) the latter often requires an accurate model of a structure, but constructing such a model can be challenging for complex structures [7] and (2) the former can be applied without a model of a structure and quantitative knowledge of the structure, such as its material properties and boundary conditions. It has been shown that curvatures in mode shapes and operating deflection shapes are more damage-sensitive than mode shapes and operating deflection shapes, as prominent local anomalies can be clearly observed in neighborhoods of damage in the the curvatures [8,9,10,11]. The use of curvatures in mode shapes for damage identification was first proposed in [8], and it was extended to curvatures of operating deflection shapes in [9]. Efforts have been made to manifest local anomalies in the curvatures and to isolate the anomalies to identify the locality and extent of damage. The use of continuous wavelet transforms was proposed and investigated in [12,13,14,15,16] to calculate the curvatures of mode shapes with high signal-to-noise ratios. The adverse effects of measurement noise and errors can be minimized by increasing the value of the scale parameter, and local anomalies corresponding to the damage can be prominently isolated. Multi-resolution operators to calculate the curvatures with high signal-to-noise ratios were proposed, where the adverse effects of measurement noise and errors can be minimized by increasing the value of a resolution parameter [17,18], and the use of polynomial fits was proposed to isolate the local anomalies when orders of the polynomial fits were properly determined [17,18]. Besides vibration characteristics, the occurrence of changes in local mass and/or stiffness, which is caused by that of damage, can lead to changes in propagating elastic waves [19,20,21,22]. Similar to mode shapes and operating deflection shapes, propagating elastic waves can undergo local intrinsic changes that occur in the neighborhood of the damage. Besides the propagation, the changes due to the damage can be captured and visualized in wavefields that are formed by wave propagation data on a predefined measurement gird. A wavefield is defined in the time and space domains, and Lamb wave features including wavenumbers and frequencies can be obtained for damage identification purposes using multidimensional Fourier transforms [22,23,24,25,26]. Zero-lag cross-correlation algorithms have been developed to process measured wavefields to identify damage in composite structures [27,28,29]. To date, there are two categories of damage identification methods: one is based on curvatures in mode shapes and operating deflection shapes and the other is based on propagating elastic waves. They have been independently studied, but their similarity and applications have not been identified and investigated.

In this work, a baseline-free structural damage identification method is developed for beam-like structures, and the method identifies locality of damage based on a new concept of curvature waveforms of propagating flexural waves. To alleviate adverse effects of measurement noise/errors in measured waveforms, a multi-resolution local-regression temporal-spatial curvature damage index (TSCDI) is defined in a pointwise manner. In the TSCDI, a multi-resolution finite difference scheme is used to calculate curvature waveforms and the use of local-regression polynomials is proposed to estimate the waveforms and associated curvature waveforms of a pseudo-pristine structure. High TSCDI values can be observed in the neighborhood of damage, and they can be inversely used to identify locality of the damage. A two-dimensional auxiliary TSCDI and a one-dimensional auxiliary damage index are proposed to further assist the identification. Numerical investigations are conducted to study the effectiveness and robustness of the developed method for different resolution parameters in the multi-resolution finite difference scheme, width parameters in the local-regression polynomials, and levels of measurement noise in waveforms. An experimental investigation was conducted on a damaged cantilever beam to validate the effectiveness of the developed method. The developed method is more damage-sensitive than the existing curvature-based methods, as it uses propagating flexural waves. Meanwhile, it is more noise-robust than the existing flexural wave-based methods, as the effects of damage are accumulatively quantified by the proposed TSCDIs bases that are calculated using each waveform.

The rest of the paper is arranged as follows. In Section 2, the developed damage identification method is described. In Section 3, the numerical investigations are presented. In Section 4, the experimental investigation is presented. Conclusions of this work are presented in Section 5.

## 2. Methodology

### 2.1. TSCDI Based on Curvature Waveforms of Propagating Flexural Waves

A propagating flexural wave of a beam-like structure can be described by $w\left(x,t\right)$, which is a function of two variables, including the spatial position *x* and time *t*. A waveform of the propagating flexural wave at *t* is defined as the instantaneous shape of the wave at *t*, and it is a function of *x*. The curvature of a waveform, referred to as the curvature waveform, of a beam-like structure is related to its instantaneous bending moment and flexural stiffness at *t*. By assuming that the dimensions, material properties and boundary conditions of the structure are time-invariant and the slope of *w* along the length of the structure is sufficiently small, the relation among the curvature waveform, bending moment and flexural stiffness of the structure can be expressed by [30]

(1) $$w^{\prime \prime }\left(x,t\right)=\frac{\partial^{2}w\left(x,t\right)}{\partial x^{2}}=\frac{M\left(x,t\right)}{EI\left(x\right)}$$

where a prime denotes the first-order partial differentiation with respect to *x*, $M\left(x,t\right)$ denotes the bending moment at *x* at *t* and $EI\left(x\right)$ denotes the flexural stiffness at *x*. When local damage occurs to the structure at $\hat{x}$, EI can be reduced at $\hat{x}$ or in the neighborhood of $\hat{x}$. As a result, the magnitude of $w^{\prime \prime }$ at $\hat{x}$ or in the neighborhood of $\hat{x}$ will be increased if *M* remains unchanged. The reduction in EI and increase of the magnitude of $w^{\prime \prime }$ are local in nature, and EI and the magnitude of $w^{\prime \prime }$ in intact areas away from the damage remain unchanged for the same *M*.

A damage index based on comparison between curvatures of mode shapes of damaged and pristine beam-like structures was proposed in [17] to identify the locality and extent of damage. Based on Equation (2), a TSCDI is proposed based on a comparison between curvature waveforms of damaged and pristine beam-like structures, and it is expressed by

(2) $$\delta \left(x,t\right)=\left|w_{dmg}^{\prime \prime }\left(x,t\right)-w_{prst}^{\prime \prime }\left(x,t\right)\right|$$

where $\left|\cdot \right|$ denotes an absolute value, and $w_{dmg}$ and $w_{prst}$ denote a waveform of the damaged structure and that of the pristine structure, respectively. Relatively high δ values can be expected in neighborhoods of the damage, and the locality and extent of the neighborhoods be inversely used to identify those of the damage.

### 2.2. Local-Regression Waveforms of Pseudo-Pristine Structure, Local-Regression TSCDI and Auxiliary TSCDI

As proposed in Section 2.1, damage can be identified based on δ in Equation (2) if $w_{dmg}$ and $w_{prst}$ are available. However, $w_{prst}$ is not always available in practice for a structure to be examined. By assuming that a pristine beam-like structure is geometrically smooth and made of materials with no stiffness and mass discontinuities, waveforms of its propagating flexural waves and associated curvature waveforms are spatially smooth at all sampled instants, and any anomalies existing in the curvature waveforms can be considered caused by damage. In [17], it was proposed that a mode shape and its curvature of a pristine beam-like structure be approximated by the use of a polynomial that fits a corresponding mode shape of a damaged beam-like structure. However, since a waveform of a propagating flexural wave of a beam-like structure can have a high order, the formulation of a solution for coefficients of a polynomial that fits the waveform spanning the length of the structure can be numerically ill-conditioned. To avoid the potential numerical ill-conditioning problem, it is proposed that a waveform of a pristine beam-like structure be approximated by the use of polynomials in a local-regression manner. A waveform of a pristine beam-like structure and its associated curvature waveform at *x* at *t* are estimated using a local-regression polynomial that fits a waveform of damaged beam-like structure in an interval. The interval is centered at *x* with a width parameter denoted by ξ that ranges between 0% and 100%, and it is described by $\left[x-\frac{\xi \Delta xm}{2},x+\frac{\xi \Delta xm}{2}\Delta x\right]$, with Δx and *m* being the distance between two neighboring measurement points and the number of measurement points on a measurement grid, respectively. The local-regression polynomial is expressed by

(3) $$w_{poly}\left(x,t\right)=\sum_{k=0}^{n\left(x,t\right)}b_{k}\left(x,t\right)x^{k}$$

where $n\left(x,t\right)$ and $b_{k}\left(x,t\right)$ denote the order and coefficients of the polynomial, respectively, for the interval centered at *x* at *t*. The coefficients $b_{k}\left(x,t\right)$ can be estimated by solving a linear equation

(4) $$A\left(x\right)b\left(x,t\right)=w\left(x,t\right)$$

where

(5) $$A\left(x\right)=\left[\begin{array}{ccccc} 1 & {\bar{x}}_{1} & {\bar{x}}_{1}^{2} & \ldots & {\bar{x}}_{1}^{n}\\ 1 & {\bar{x}}_{2} & {\bar{x}}_{2}^{2} & \ldots & {\bar{x}}_{2}^{n}\\ \vdots & \vdots & \vdots & \ddots & \vdots \\ 1 & {\bar{x}}_{m} & {\bar{x}}_{m}^{2} & \ldots & {\bar{x}}_{m}^{n} \end{array}\right]$$

is an $m\times \left(n+1\right)$ Vandermonde matrix, in which ${\bar{x}}_{j}=\frac{2x_{j}-2x}{\xi \Delta xm}$ is the *j*-th normalized *x*-coordinate corresponding to the *j*-th discrete *x*-coordinate in the interval,

(6) $$b\left(x,t\right)={\left\{\begin{array}{cccc} b_{0}\left(x,t\right), & b_{1}\left(x,t\right), & \ldots , & b_{n}\left(x,t\right) \end{array}\right\}}^{T}$$

is an $\left(n+1\right)$-dimensional vector, in which the superscript T denotes the transpose of a matrix, and

(7) $$w\left(x,t\right)={\left\{\begin{array}{cccc} w\left(x-\frac{\xi \Delta xm}{2},t\right), & w\left(x-\frac{\xi \Delta xm}{2}+\Delta x,t\right), & \ldots , & w\left(x+\frac{\xi \Delta xm}{2},t\right) \end{array}\right\}}^{T}$$

is an *m*-dimensional vector. The normalization for ${\bar{x}}_{j}$ is performed using the “center and scale” technique [31] such that ${\bar{x}}_{j}$ in Equation (5) has the following properties: ${\bar{x}}_{j}\in \left[-1,1\right]$, ${\bar{x}}_{j}=-1$ if $x=x-\frac{\xi \Delta xm}{2}$, and ${\bar{x}}_{j}=1$ if $x=x+\frac{\xi \Delta xm}{2}$. Note that *m* is usually sufficiently larger than *n*, i.e., m≫n, and the linear equation in Equation (4) becomes over-determined. A solution to the linear equation in Equation (4) can be calculated by

(8) $$b\left(x,t\right)=A^{†}\left(x\right)w\left(x,t\right)$$

where the superscript † denotes the Moore–Penrose inverse of a matrix. With a calculated $b\left(x,t\right)$ from Equation (8), the value of $w_{poly}$ at $x_{j}$ in the interval can be estimated by

(9) $$w_{poly}\left(x_{j},t\right)=A_{j}b\left(x,t\right)$$

where

(10) $$A_{j}=\left\{\begin{array}{ccccc} 1, & {\bar{x}}_{j}, & {\bar{x}}_{j}^{2}, & \ldots , & {\bar{x}}_{j}^{n} \end{array}\right\}$$

is the *j*-th row of A in Equation (5).

The level of approximation of a waveform from the polynomial in Equation (3) to $w_{dmg}$ for the interval $\left[x-\frac{\xi \Delta xm}{2},x_{r}+\frac{\xi \Delta xm}{2}\right]$ can be quantified by a modal assurance criterion value, which is expressed by

(11) $$\mathrm{MAC}\left(w_{poly}\left(x,t\right),w_{dmg}\left(x,t\right)\right)=\frac{{∥w_{poly}^{H}\left(x,t\right)w_{dmg}\left(x,t\right)∥}^{2}}{{∥w_{poly}\left(x,t\right)∥}^{2}{∥w_{dmg}\left(x,t\right)∥}^{2}}\times 100\%$$

where

(12) $$w_{poly}\left(x,t\right)={\left\{w_{poly}\left(x-\frac{\xi \Delta xm}{2},t\right),w_{poly}\left(x-\frac{\xi \Delta xm}{2}+\Delta x,t\right),\ldots ,w_{poly}\left(x+\frac{\xi \Delta xm}{2},t\right)\right\}}^{T}$$

and

(13) $$w_{dmg}\left(x,t\right)={\left\{w_{dmg}\left(x-\frac{\xi \Delta xm}{2},t\right),w_{dmg}\left(x-\frac{\xi \Delta xm}{2}+\Delta x,t\right),\ldots ,w_{dmg}\left(x+\frac{\xi \Delta xm}{2},t\right)\right\}}^{T}$$

are *m*-dimensional vectors, the superscript H denotes the conjugate transpose of a matrix and $∥\cdot ∥$ denotes the $L_{2}$-norm of a complex scalar or vector. It is shown in [17] that the level of approximation between a mode shape and that from a polynomial that fits the mode shape is related to the order of the polynomial; the higher the order, the higher the level of approximation. Similarly, the approximation of $w_{poly}$ to $w_{dmg}$ is related to *n* in Equation (3). An appropriate value of *n* is determined to be two plus the smallest order with which the value of MAC is larger than 90%, and an extra two is included in the determination to preserve the degree of a curvature waveform, since its calculation incurs a second-order partial differentiation with respect to *x*.

By combing the TSCDI δ in Equation (2) and local-regression waveform $w_{poly}$ in Equation (3), a local-regression TSCDI is proposed for damage identification solely based on $w_{dmg}$, and it is expressed by

(14) $$\delta_{l-r}\left(x,t\right)=\left|w_{dmg}^{\prime \prime }\left(x,t\right)-w{}_{poly}^{\prime \prime }\left(x,t\right)\right|$$

The local-regression TSCDI $\delta_{l-r}$ can serve as an indicator of the locality and extent of structural damage and $\delta_{l-r}$ is advantageous over δ in Equation (2) as $\delta_{l-r}$ does not require any knowledge of $w_{prst}$. Based on $\delta_{l-r}$, an auxiliary TSCDI is proposed to offer an indication of the locality and extent of the damage, and it is expressed by

(15) $$\tilde{\delta }\left(x\right)=\frac{\int_{t_{0}}^{t_{1}}\delta_{l-r}\left(x,t\right)dt}{\max_{\frac{x}{L}\in \left[0,1\right]}\left(\int_{t_{0}}^{t_{1}}\delta_{l-r}\left(x,t\right)dt\right)}$$

where $\max_{\frac{x}{L}\in \left[0,1\right]}\left(\int_{t_{0}}^{t_{1}}\delta_{l-r}\left(x,t\right)dt\right)$ denotes the maximum value of $\int_{t_{0}}^{t_{1}}\delta_{l-r}\left(x,t\right)dt$ for $x\in \left[0,L\right]$, in which $t_{0}$ and $t_{1}$ denote the beginning and ending instants of measured *w*, respectively, and *L* is the length of measured *w*. Note that $\tilde{\delta }$ ranges between 0 and 1. The locality and extent of the damage can be identified in neighborhoods with high $\tilde{\delta }$ values.

### 2.3. Multi-Resolution Local-Regressioon TSCDIs, Multi-Resolution Auxiliary TSCDIs and Auxiliary CDIs

There are various numerical schemes to calculate $w^{\prime \prime }$ with different orders of accuracy [32]. One of the most commonly used schemes is the central finite difference scheme, which has the first-order accuracy, and it can be expressed by

(16) $$w^{\prime \prime }\left(x,t\right)=\frac{w\left(x+\Delta x,t\right)-2w\left(x,t\right)+w\left(x-\Delta x,t\right)}{\Delta x^{2}}$$

The value of Δx is determined by the spatial density of the discrete *x* and it is uniform for all discrete *x* if they are equally spaced. When a waveform is contaminated by measurement noise and errors, it can be expressed by

(17) $$\tilde{w}\left(x,t\right)=w\left(x,t\right)+\epsilon \left(x,t\right)$$

where $\epsilon \left(x,t\right)$ denotes the measurement noise and error. Applying the finite difference scheme in Equation (16) for $\tilde{w}$ by replacing *w* with $\tilde{w}$ yields

(18) $$\begin{array}{ccc} {\tilde{w}}^{\prime \prime }\left(x,t\right) & = & \frac{\tilde{w}\left(x+\Delta x,t\right)-2\tilde{w}\left(x,t\right)+\tilde{w}\left(x-\Delta x,t\right)}{\Delta x^{2}}\\ \; & = & \frac{w\left(x+\Delta x,t\right)+\epsilon \left(x+\Delta x,t\right)-2w\left(x,t\right)-2\epsilon \left(x,t\right)+w\left(x-\Delta x,t\right)+\epsilon \left(x-\Delta x,t\right)}{\Delta x^{2}}\\ \; & = & w^{\prime \prime }\left(x,t\right)+\epsilon^{\prime \prime }\left(x,t\right) \end{array}$$

where

(19) $$\epsilon^{\prime \prime }\left(x,t\right)=\frac{\epsilon \left(x+\Delta x,t\right)-2\epsilon \left(x,t\right)+\epsilon \left(x-\Delta x,t\right)}{\Delta x^{2}}$$

numerically calculates the second-order differentiation of ϵ with respect to *x* using the finite difference scheme in Equation (16). By assuming that ϵ is zero-mean with a standard deviation σ and the value of σ is independent of *x*, the numerator of Equation (19), i.e., $\epsilon \left(x+\Delta x,t\right)-2\epsilon \left(x,t\right)+\epsilon \left(x-\Delta x,t\right)$ is zero-mean, with a standard deviation that can be expressed by

(20) $$\sigma_{\partial^{2}\epsilon }=2\sigma$$

and the value of $\sigma_{\partial^{2}\epsilon }$ is also independent of *x*. Hence $\epsilon^{\prime \prime }$ in Equation (19) is zero-mean, with a standard deviation that can be expressed by

(21) $$\sigma_{\epsilon^{\prime \prime }}=\frac{2\sigma }{\Delta x^{2}}$$

The smaller the value of Δx, the larger the value of $\sigma_{\epsilon^{\prime \prime }}$. When applying the finite difference scheme in Equation (16) with noise-free *w* and a sufficiently small Δx, resulted $w^{\prime \prime }$ can converge to exact values of $w^{\prime \prime }$, but when *w* is contaminated by ϵ, its curvature, i.e., ${\tilde{w}}^{\prime \prime }$, can be dominated by $\epsilon^{\prime \prime }$ with adversely amplified amplitudes due to the sufficiently small Δx, according to Equation (16).

To alleviate the adverse amplitude amplification of $\epsilon^{\prime \prime }$ when calculating $w^{\prime \prime }$, a multi-resolution finite difference scheme is proposed in Ref. [17], and the scheme is expressed by

(22) $$w_{r}^{\prime \prime }\left(x,t\right)=\frac{w\left(x+r\Delta x,t\right)-2w\left(x,t\right)+w\left(x-r\Delta x,t\right)}{{\left(r\Delta x\right)}^{2}}$$

where *r* denotes an integer resolution parameter. Applying the scheme in Equation (22) to $\tilde{w}$ yields

(23) $$\begin{array}{ccc} {\tilde{w}}_{r}^{\prime \prime }\left(x,t\right) & = & \frac{\tilde{w}\left(x+r\Delta x,t\right)-2\tilde{w}\left(x,t\right)+\tilde{w}\left(x-r\Delta x,t\right)}{{\left(r\Delta x\right)}^{2}}\\ \; & = & \frac{w\left(x+r\Delta x,t\right)+\epsilon \left(x+r\Delta x,t\right)-2w\left(x,t\right)-2\epsilon \left(x,t\right)+w\left(x-r\Delta x,t\right)+\epsilon \left(x-r\Delta x,t\right)}{{\left(r\Delta x\right)}^{2}}\\ \; & = & w_{r}^{\prime \prime }\left(x,t\right)+\epsilon_{r}^{\prime \prime }\left(x,t\right) \end{array}$$

where

(24) $$w_{r}^{\prime \prime }\left(x,t\right)=\frac{w\left(x+r\Delta x,t\right)-2w\left(x,t\right)+w\left(x-r\Delta x,t\right)}{{\left(r\Delta x\right)}^{2}}$$

and

(25) $$\epsilon_{r}^{\prime \prime }\left(x,t\right)=\frac{\epsilon \left(x+r\Delta x,t\right)-2\epsilon \left(x,t\right)+\epsilon \left(x-r\Delta x,t\right)}{{\left(r\Delta x\right)}^{2}}$$

The standard deviation of $\epsilon_{r}^{\prime \prime }$ in Equation (25) can be expressed by

(26) $$\sigma_{\epsilon_{r}^{\prime \prime }}=\frac{2\sigma }{{\left(r\Delta x\right)}^{2}}$$

Similar to $\sigma_{\epsilon^{\prime \prime }}$ in Equation (21), $\sigma_{\epsilon_{r}^{\prime \prime }}$ is independent of *x*; more importnatly, $\sigma_{\epsilon_{r}^{\prime \prime }}$ is inversely related to $r^{2}$. Hence, the larger the value of *r*, the smaller the amplitude magnification of $\epsilon_{r}^{\prime \prime }$ in ${\tilde{w}}_{r}^{\prime \prime }$, and amplitude amplification of $\epsilon^{\prime \prime }$ can be reduced by increasing *r* in the multi-resolution finite difference scheme in Equation (23).

Based on the multi-resolution finite difference scheme in Equation (23), a multi-resolution local-regression TSCDI is proposed, and it is expressed by

(27) $$\delta_{l-r,r}\left(x,t\right)=\left|w_{dmg,r}^{\prime \prime }\left(x,t\right)-w_{poly,r}^{\prime \prime }\left(x,t\right)\right|$$

where $w_{dmg,r}^{\prime \prime }$ and $w_{poly,r}^{\prime \prime }$ denote a curvature waveform of a damaged structure and that from a polynomial, respectively, at *x* at *t*, and they are calculated using the multi-resolution finite difference scheme in Equation (22). An auxiliary CDI is proposed to further assist with the damage identification based on $\delta_{l-r,r}$ with multiple *r*, and it is expressed by

(28) $$\chi \left(x\right)=\sum_{r=1}^{R}{\left|{\tilde{\delta }}_{r}\left(x\right)\right|}^{2}$$

where *R* denotes the maximum resolution value in the multi-resolution finite difference scheme in $\delta_{l-r,r}$ and

(29) $${\tilde{\delta }}_{r}=\frac{\int_{t_{0}}^{t_{1}}\delta_{l-r,r}\left(x,t\right)dt}{\max_{\frac{x}{L}\in \left[0,1\right]}\left(\int_{t_{0}}^{t_{1}}\delta_{l-r,r}\left(x,t\right)dt\right)}$$

denotes a multi-resolution auxiliary TSCDI with *r*, and ${\tilde{\delta }}_{r}\in \left[0,1\right]$. Damage can be identified in neighborhoods with consistently high ${\tilde{\delta }}_{r}$ values with multiple *r* and those with high χ values.

A step-by-step description of the proposed damage identification method is described below:

Step 1. Waveforms of a propagating flexural wave $w_{dmg}$ is measured at equally spaced discrete measurement points assigned along the length of a damaged beam-like structure;
Step 2. The multi-resolution local-regression TSCDI $\delta_{l-r,r}$ is calculated for the waveform at each discrete *t* with multiple *r* measured in Step 1;
  2.1 The value of ξ is determined and $w_{poly}$ is calculated for each discrete *x* based on Equations (3) through (9);
  2.2 Curvature waveforms $w_{dmg,r}^{\prime \prime }$ and $w_{poly,r}^{\prime \prime }$ are calculated using the multi-resolution finite difference scheme in Equation (23) with multiple *r*;
  2.3 TSCDIs $\delta_{l-r,r}$ in Equation (27) are calculated using $w_{dmg,r}^{\prime \prime }$ and $w_{poly,r}^{\prime \prime }$ obtained in Step 2.2;
Step 3. The multi-resolution auxiliary TSCDI ${\tilde{\delta }}_{r}$ in Equation (29) and auxiliary CDI χ in Equation (28) are calculated using $\delta_{l-r,r}$ obtained in Step 2;
Step 4. Identify damage in neighborhoods with consistently high ${\tilde{\delta }}_{r}$ values with different *r* and those with high χ values obtained in Step 3.

## 3. Numerical Investigation

### 3.1. Finite Element Models of Damaged and Pristine Beams

**Figure 1:**
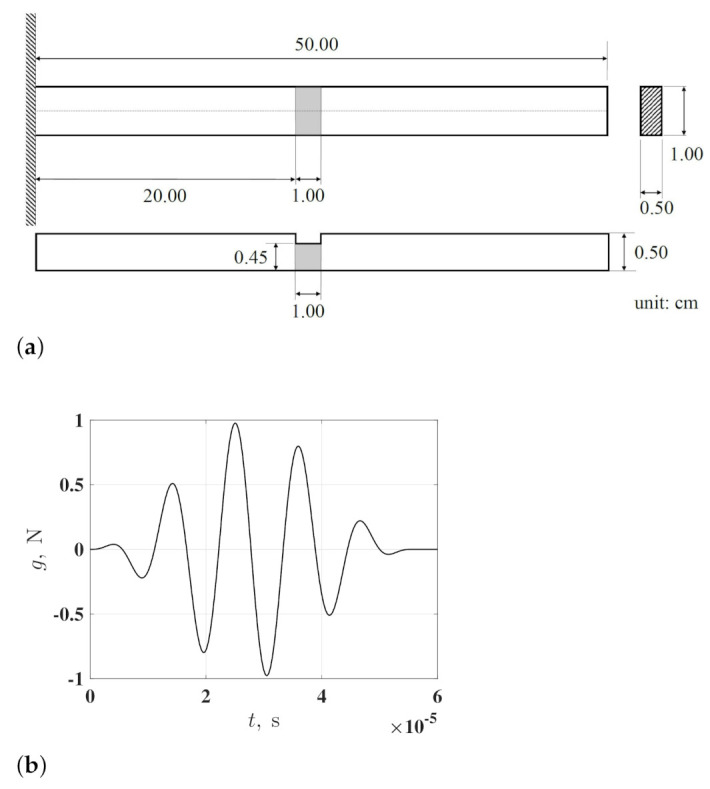
(**a**) Dimensions of a damaged cantilever beam with damage in the form of a one-sided thickness reduction area and (**b**) an excitation force in the form of a 5-count wave packet.

A finite element model of a damaged cantilever beam and that of a pristine cantilever beam are constructed using linear eight-node brick (C3D8R) elements to numerically investigate the proposed damage identification method. The dimensions and boundary conditions of the damaged beam are described in Figure 1a and the damage is in the form of a one-sided thickness reduction area. The damaged beam is made of aluminum with a mass density of 2700 kg/m^3^, an elastic modulus of 69 GPa and Poisson’s ratio of 0.33. The pristine beam has the same dimensions, boundary conditions and material properties as those of the damaged beam. The damaged and pristine beams are both under zero initial conditions and subject to the same excitation force applied to their free ends. The force can be analytically expressed by an $N_{c}$-count wave packet

(30) $$g\left(t\right)=A\left(H\left(t\right)-H\left(t-\frac{N_{c}}{f_{c}}\right)\right)\left(1-\cos\left(\frac{2\pi f_{c}t}{N_{c}}\right)\right)\sin\left(2\pi f_{c}t\right)$$

where *A* is a parameter that determines the amplitude of *g*, *H* is Heaviside function, which can be expressed by

(31) $$H\left(t\right)=\left\{\begin{array}{ccc} 1 & , & t\geq 0\\ 0 & , & t<0 \end{array}\right.$$

and $f_{c}$ denotes the central frequency of the force. In this investigation, A=0.5 N, $N_{c}=5$ and $f_{c}=90$ kHz, which makes the excitation force a 5-count wave packet with a central frequency of 90 kHz, as shown in Figure 1b.

### 3.2. Waveforms of Damaged, Pristine and Pseudo-Pristine Beams and Curvature Waveforms

**Figure 2:**
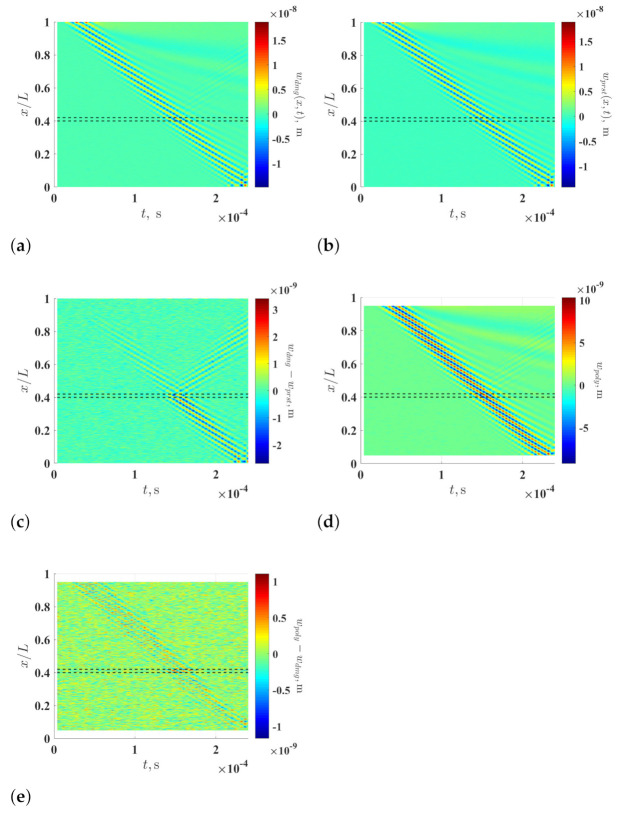
Noise-contaminated waveforms of the propagating flexural waves of the (**a**) damaged and (**b**) pristine beams from the finite element models, (**c**) the difference between the waveforms in (**a**,**b**), (**d**) waveforms of the propagating flexural wave of the pseudo-pristine beam and (**e**) the difference between the waveforms in (**a**,**d**). The locality and extent of the damage are indicated by two dashed lines.

Waveforms of propagating flexural waves of the damaged and pristine beams, which are caused by the excitation, are obtained at 1001 measurement points that are evenly distributed along the lengths of the beams with a sampling frequency of 250 kHz for the first $2.4\times 10^{-4}$ s. To simulate measurement noise, white Gaussian noise is added to the response of each measurement point of the two beams so that it has a signal-to-noise ratio of 20 dB. The signal-to-noise ratio is defined as the ratio between powers of the response of a measurement point and the added noise. Noise-contaminated waveforms of the propagating flexural waves of the damaged and pristine beams are shown in Figure 2a,b, respectively, and their difference is shown in Figure 2c. Although the waveforms are contaminated by the simulated measurement noise, they compare well with each other until the wave of the damaged beam reaches the damage, where a reflection of the wave occurs, and the amplitude of their difference increases after the wave passes the damage of the damaged beam. Waveforms of the propagating flexural wave of the pseudo-pristine beam, which correspond to the noise-contaminated waveforms of the damaged beam, are shown in Figure 2d, and their difference with the contaminated waveforms of the damaged beam are shown in Figure 2e. It can be seen that the waveforms of the damaged and pseudo-pristine beams compare well through the propagation, since no wave reflection occurs and the amplitude of the difference between the two waves does not increase after the wave passes the damage in the damaged beam.

**Figure 3:**
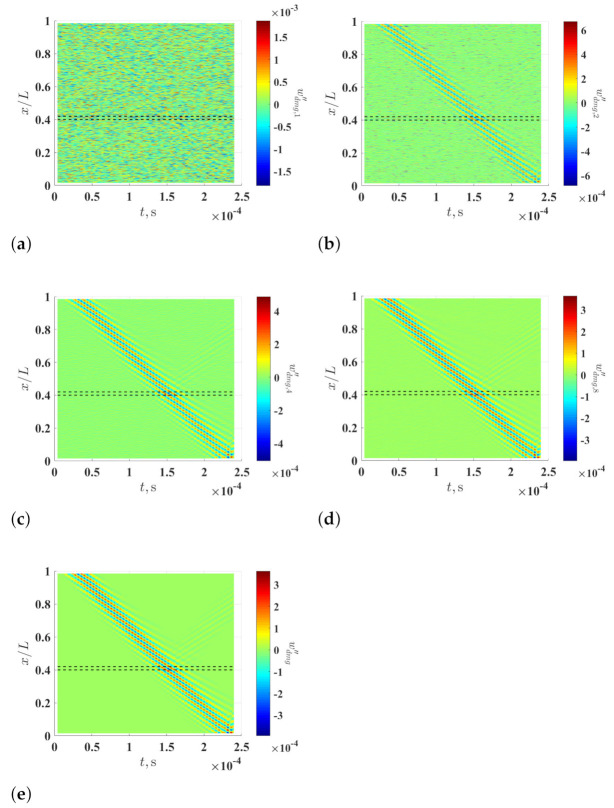
Curvature waveforms of the noise-contaminated wave of the damaged beam, which are calculated using the multi-resolution finite difference scheme with (**a**) r=1, (**b**) r=2, (**c**) r=4 and (**d**) r=8, and (**e**) curvature waveforms of the noise-free wave of the damaged, which are calculated using the multi-resolution finite difference scheme with r=8. Locality and extent of the damage are indicated by two dashed lines.

Curvature waveforms associated with the noise-contaminated waveforms of the damaged beam, which are calculated using the multi-resolution finite difference scheme with r=1, 2, 4 and 8, are shown in Figure 3a–d, respectively. It can be seen that noise levels of the curvature waveforms associated with the noise-contaminated waveforms are lowered by increasing *r* value. Curvature waveforms of the noise-free waveforms of the damaged beam, which are calculated with the multi-resolution finite difference scheme with r=8, are shown in Figure 3e. By comparing Figure 3d,e, the curvature waveforms associated with the noise-contaminated waveforms well approximate those associated with the noise-free waveforms. It is verified that the adverse effects of measurement noise on calculation of curvature waveforms can be alleviated by the multi-resolution scheme, while the accuracy of calculated curvature waveforms can be retained.

### 3.3. Damage Identification Results

**Figure 4:**
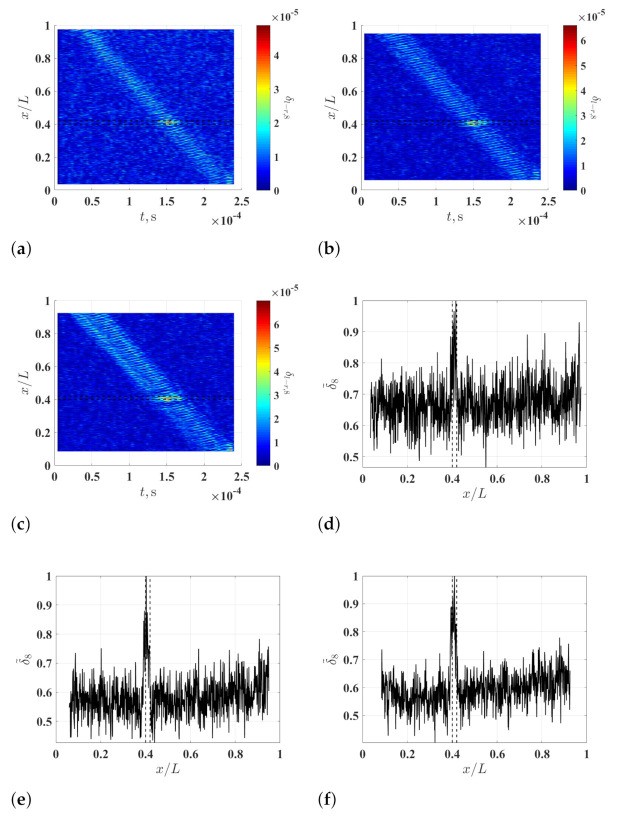
Multi-resolution local-regression TSCDI $\delta_{l-r,r}$ associated with the noise-contaminated wave of the damaged beam with r=8 and (**a**) ξ=5%, (**b**) ξ=10% and (**c**) ξ=15%, and multi-resolution auxiliary TSCDI ${\tilde{\delta }}_{r}$ with associated with the noise-contaminated wave of the damaged beam with r=8 and (**d**) ξ=5%, (**e**) ξ=10% and (**f**) ξ=15%. Locality and extent of the damage are indicated by two dashed lines.

**Figure 5:**
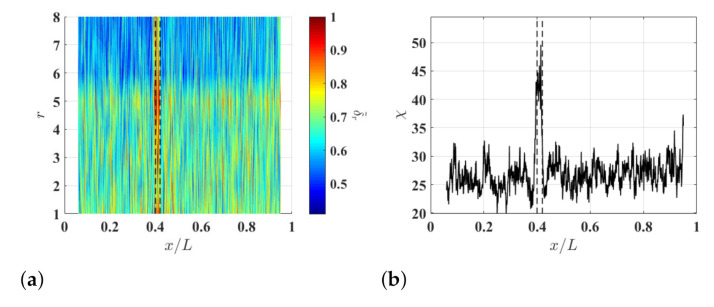
(**a**) Multi-resolution auxiliary TSCDI ${\tilde{\delta }}_{r}$ associated with $\delta_{l-r,r}$ with r=1,2,…,8 and ξ=10% and (**b**) auxiliary CDI χ associated with ${\tilde{\delta }}_{r}$ in (**a**). Locality and extent of the damage are indicated by two dashed lines.

To study the effects of ξ on the damage identification method, $\delta_{l-r,r}$ associated with the noise-contaminated waveforms of the damaged beam is calculated with r=8 and different ξ values. Resulting $\delta_{l-r,8}$ with ξ=5%, ξ=10% and ξ=15% are shown in Figure 4a–c, respectively. Relatively high $\delta_{l-r,8}$ values can be observed in the neighborhood of the damage and they correspond to changes in curvature waveforms caused by the damage. Multi-solution auxiliary TSCDI ${\tilde{\delta }}_{r}$ with r=8, i.e., ${\tilde{\delta }}_{8}$, associated with $\delta_{l-r,8}$ with ξ=5%, ξ=10% and ξ=15% are shown in Figure 4d–f, respectively, and the locality and extent of the damage can be identified based on the three ${\tilde{\delta }}_{8}$. Further, it can be seen that increasing ξ from 5% to 10% can improve identification results by lowering the noise floor of ${\tilde{\delta }}_{r}$, but the improvement becomes insignificant when ξ is increased from 10% to 15%, which indicates that ${\tilde{\delta }}_{r}$ can converge when ξ is large enough. In practice, one can determine an appropriate ξ value by increasing ξ until convergent ${\tilde{\delta }}_{r}$ is obtained. Multi-resolution auxiliary TSCDI ${\tilde{\delta }}_{r}$ associated with $\delta_{l-r,r}$ with r=1,2,…,8 and ξ=10% is shown in Figure 5a. When r<6, relatively high noise floors can be observed in $\delta_{l-r,r}$, and when r≥6, the noise floor of $\delta_{l-r,r}$ becomes lower. Auxiliary CDI χ associated with $\delta_{l-r,r}$ in Figure 5a is shown in Figure 5b, where the locality and extent of the damage can be clearly identified.

**Figure 6:**
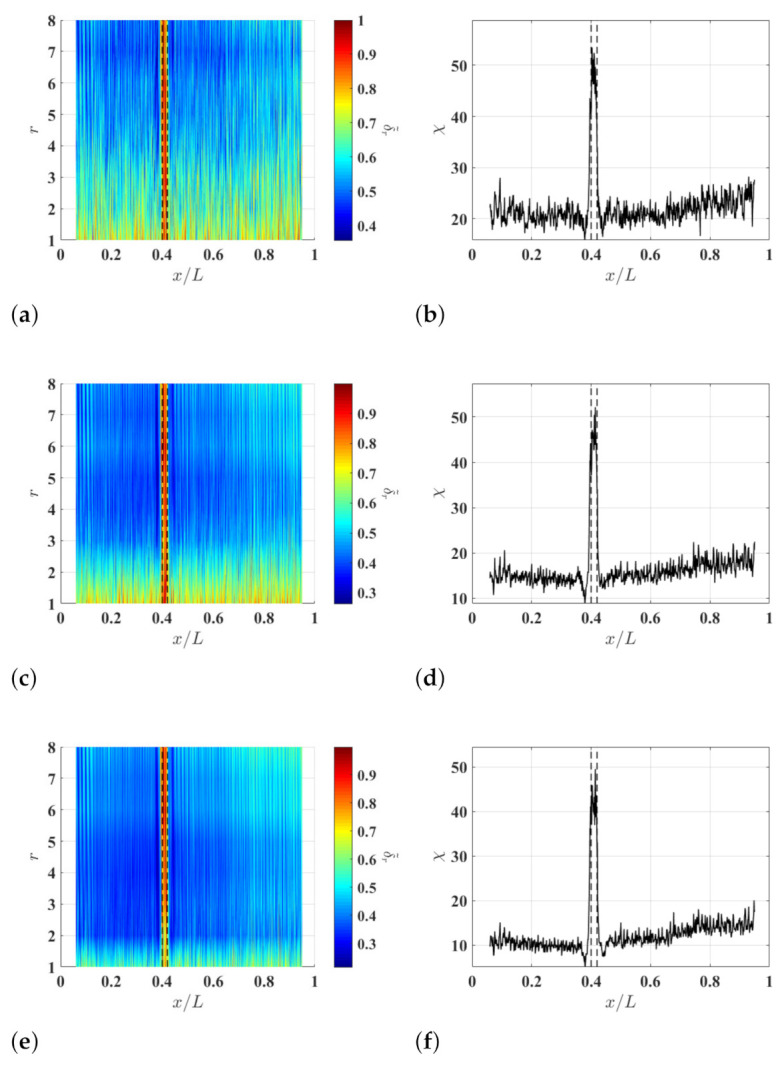
(**a**) Multi-resolution auxiliary TSCDI ${\tilde{\delta }}_{r}$ associated with the noise-contaminated waves with signal-to-noise ratios of (**a**) 30 db, (**b**) 40 db and (**c**) 50 db, which are calculated with r=1,2,…,8 and ξ=10%, and auxiliary CDI χ associated with (**d**) ${\tilde{\delta }}_{r}$ in (**a**), (**e**) ${\tilde{\delta }}_{r}$ in (**a**), and (**f**) ${\tilde{\delta }}_{r}$ in (**c**). Locality and extent of the damage are indicated by two dashed lines.

To study the effects of measurement noise on the damage identification method, different levels of measurement noise are added to the noise-free responses of the beam so that they have signal-to-noise ratios of 30 db, 40 db and 50 db, and noise-contaminated waveforms with the different signal-to-noise ratios are obtained. Multi-resolution auxiliary TSCDI ${\tilde{\delta }}_{r}$ and associated auxiliary CDI χ, which correspond to the noise-contaminated waveforms, are calculated with r=1,2,…,8 and ξ=10% and shown in Figure 6. By comparing the resulting ${\tilde{\delta }}_{r}$ corresponding to the waveforms with the different signal-to-noise ratios, it can be seen that a higher *r* value corresponds to a lower noise floor of ${\tilde{\delta }}_{r}$, which further verifies the capability of the multi-resolution finite difference scheme for lowering the measurement noise amplification of curvature waveforms. By comparing the resulting χ corresponding to the waveforms with the different signal-to-noise ratios, it can be seen that the noise floor of χ is lower for waveforms with a higher signal-to-noise ratio.

## 4. Experimental Investigation

### 4.1. Experimental Setup

**Figure 7:**
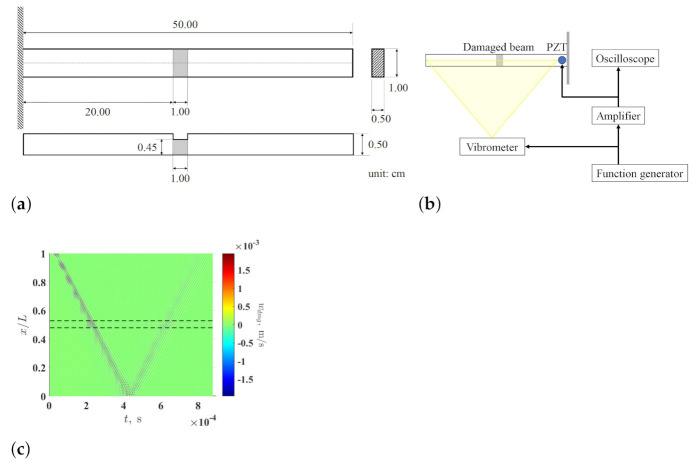
(**a**) Dimensions of a tested damaged cantilever beam with damage in the form of a one-sided thickness reduction area, (**b**) a schematic of the experimental setup for exciting and measuring a propagating flexural wave of the damaged beam, and (**c**) the denoised measured propagating flexural wave of the damaged beam, where the locality and extent of the damage are indicated by two dashed lines.

An aluminum damaged cantilever beam was prepared and tested to experimentally investigate the proposed damage identification method. The beam had damage in the form of a one-sided thickness reduction area. The dimensions of the beam are shown in Figure 7a and a schematic of the experimental setup is shown in Figure 7b: a lead-zirconate-titanate (PZT) actuator was glued to the damaged surface of the beam to generate excitation in the form of a 5-count wave packet with a central frequency of 90 kHz. The excitation signal to the actuator was generated by a function generator, Tektronix AFG3022C; the peak-to-peak amplitude of the excitation signal was amplified to 50 V by a voltage amplifier, Krohn-Hite 7500, and an oscilloscope; Tektronix TBS2104 was used to monitor the amplified signal. A scanning laser Doppler vibrometer, Polytec PSV-500-HV, was used to measure flexural responses of 3585 measurement points that were evenly distributed along a scan line assigned to the beam. For each measurement point, flexural response was measured and averaged ten times to improve its signal-to-noise ratio, and the measured responses were aligned to form a measured propagating flexural wave. A numerical denoising technique, which uses weighted quadratic least squares and a second-order polynomial model, was applied to improve the signal-to-noise ratios of the waveforms of the measured wave at each sampled instant. In the denoising technique, the weighted quadratic least square is calculated at the measurement point within an interval that consists of a certain number of its neighboring measurement points, which was 0.5% in this investigation. The denoised propagating flexural wave is denoted by $w_{\mathrm{meas}}$ and shown in Figure 7c.

### 4.2. Damage Identification Results

**Figure 8:**
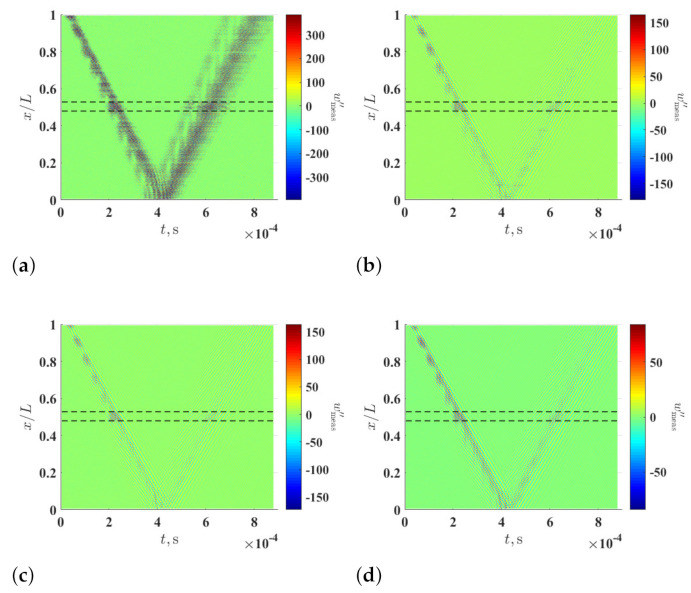
Multi-resolution curvature waveforms associated with the measured wave of the damaged beam with (**a**) r=1, (**b**) r=8, (**c**) r=16 and (**d**) r=32. Locality and extent of the damage are indicated by two dashed lines.

**Figure 9:**
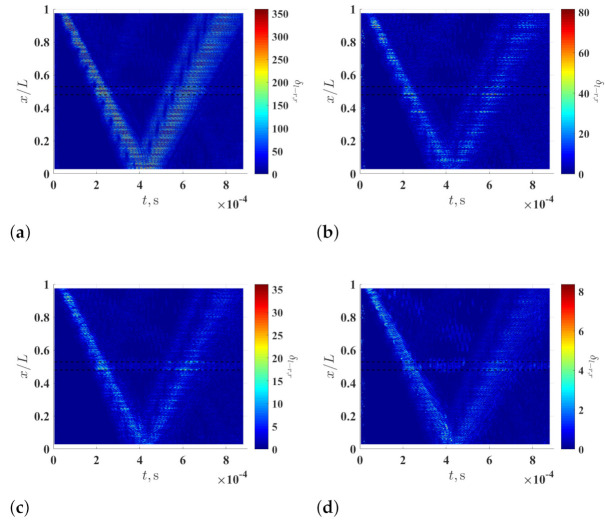
Multi-resolution local-regression TSCDI $\delta_{l-r,r}$ associated with the measured wave of the damaged beam with (**a**) r=1, (**b**) r=8, (**c**) r=16 and (**d**) r=32 and ξ=5%. Locality and extent of the damage are indicated by two dashed lines.

**Figure 10:**
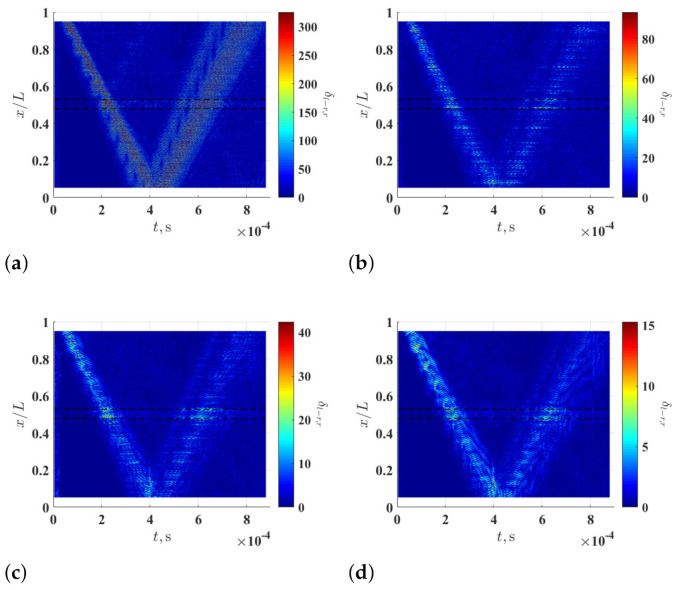
Multi-resolution local-regression TSCDI $\delta_{l-r,r}$ associated with the measured wave of the damaged beam with (**a**) r=1, (**b**) r=8, (**c**) r=16 and (**d**) r=32 and ξ=10%. Locality and extent of the damage are indicated by two dashed lines.

**Figure 11:**
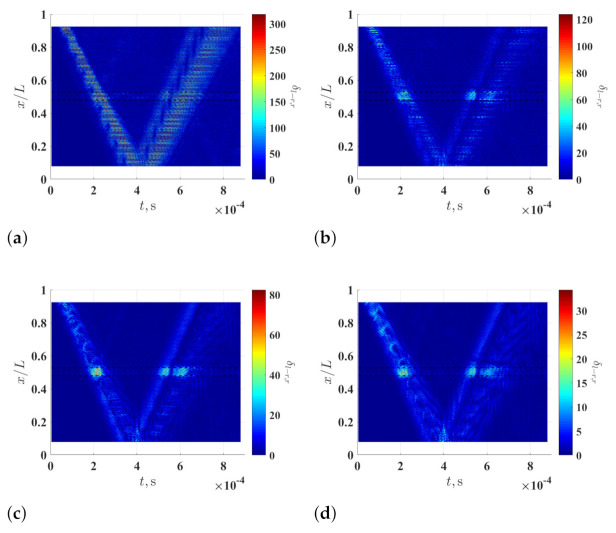
Multi-resolution local-regression TSCDI $\delta_{l-r,r}$ associated with the measured wave of the damaged beam with (**a**) r=1, (**b**) r=8, (**c**) r=16 and (**d**) r=32 and ξ=15%. Locality and extent of the damage are indicated by two dashed lines.

Figure 8a–d shows curvature waveforms of $w_{\mathrm{meas}}$, i.e., $w_{\mathrm{meas}}^{\prime \prime }$, which are calculated using the multi-resolution finite difference scheme with r=1, 8, 16 and 32, respectively. Similar to the observation in the numerical investigation, anomalies that are related to the damage cannot be directly seen in $w_{\mathrm{meas}}^{\prime \prime }$, and the signal-to-noise ratio of $w_{\mathrm{meas}}^{\prime \prime }$ can be increased by increasing *r*. Multi-resolution local-regression TSCDI $\delta_{l-r,r}$ associated with $w_{\mathrm{meas}}^{\prime \prime }$ is shown in Figure 9, where r=1, 8, 16 and 32 and ξ=5%, and those associated with ξ=10% and ξ=15% are shown in Figure 10 and Figure 11, respectively. In Figure 9a, Figure 10a and Figure 11a, the damage cannot be identified since $w_{\mathrm{meas}}^{\prime \prime }$ is dominated by amplified measurement noise with r=1 in the multi-resolution finite difference scheme. In Figure 9b–d and Figure 10b–d, where ξ=5% and ξ=10%, the damage still cannot be identified, even with increased *r* values in the multi-resolution finite difference scheme. When ξ is increased to 15%, the damage can be clearly and consistently identified in neighborhoods of high $\delta_{l-r,r}$ values with r=8, 16 and 32, as shown in Figure 9b,c, respectively.

**Figure 12:**
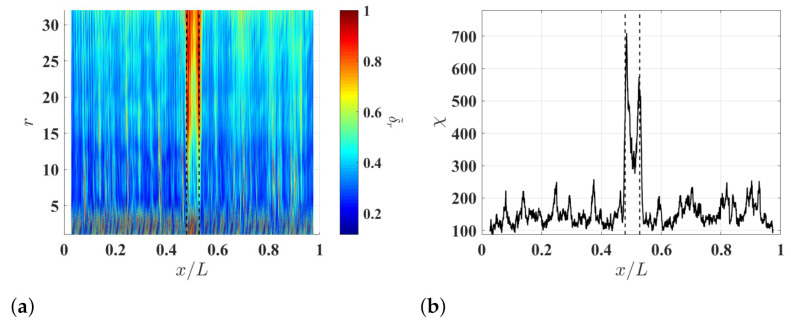
(**a**) Multi-resolution auxiliary TSCDI ${\tilde{\delta }}_{r}$ associated with $\delta_{l-r,r}$ with r=1,2,…,32 and ξ=5% and (**b**) the auxiliary CDI χ associated with ${\tilde{\delta }}_{r}$ in (**a**). Locality and extent of the damage are indicated by two dashed lines.

**Figure 13:**
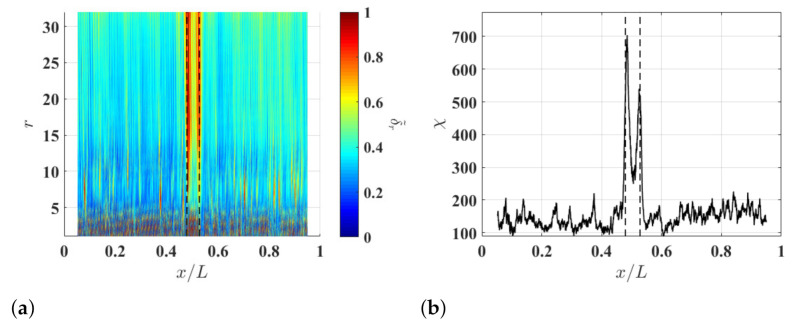
(**a**) Multi-resolution auxiliary TSCDI ${\tilde{\delta }}_{r}$ associated with $\delta_{l-r,r}$ with r=1,2,…,32 and ξ=10% and (**b**) the auxiliary CDI χ associated with ${\tilde{\delta }}_{r}$ in (**a**). Locality and extent of the damage are indicated by two dashed lines.

**Figure 14:**
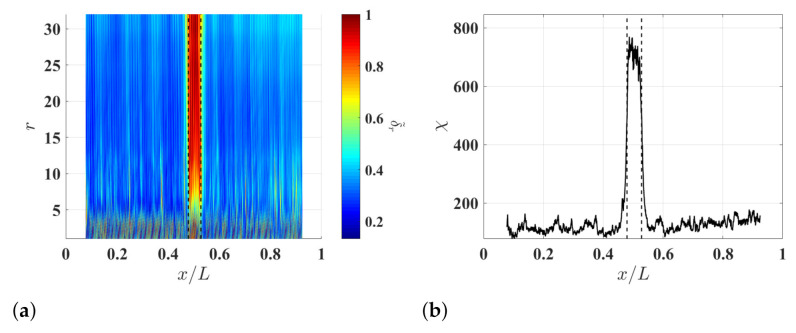
(**a**) Multi-resolution auxiliary TSCDI ${\tilde{\delta }}_{r}$ associated with $\delta_{l-r,r}$ with r=1,2,…,32 and ξ=15% and (**b**) the auxiliary CDI χ associated with ${\tilde{\delta }}_{r}$ in (**a**). Locality and extent of the damage are indicated by two dashed lines.

Multi-resolution auxiliary TSCDI ${\tilde{\delta }}_{r}$ associated with $\delta_{l-r,r}$ is calculated with ξ=5%, ξ=10% and ξ=15% are shown in Figure 12a, Figure 13a and Figure 14a, respectively, where r=1,2,…32. When r≤5, relatively high noise floors can be observed in the three ${\tilde{\delta }}_{r}$ and the effects of the damage on the curvature waveforms are masked by the amplified measurement noise in $w_{\mathrm{meas}}^{\prime \prime }$. When r≥6, the noise floors of ${\tilde{\delta }}_{r}$ become lower, and the effects of the damage on the curvature waveforms can be consistently and clearly identified in neighborhoods of high ${\tilde{\delta }}_{r}$ values. Auxiliary CDI χ associated with $\delta_{l-r,r}$ in Figure 12a, Figure 13a and Figure 14a are shown in Figure 12b, Figure 13b and Figure 14b, respectively. It can be seen that the damage can be identified in the neighborhood of high χ values and the noise floors of χ are slightly lowered by increasing ξ. More importantly, the edges of the damage can be clearly identified in the neighborhood of high χ values with ξ=5% and ξ=10%, though the damage cannot be clearly identified in associated $\delta_{l-r,r}$.

## 5. Conclusions

A baseline-free structural damage identification method is developed for beam-like structures using curvature waveforms of propagating flexural waves. The identification does not require use of quantitative information of the structures to be examined, such as material properties and boundary conditions, if the structures are geometrically smooth and made of materials that have no mass and stiffness discontinuities. Curvature waveforms are calculated using a multi-resolution finite difference scheme, and it is shown that the scheme can accurately estimate curvature waveforms and alleviate the adverse effects of amplified measurement noise. Waveforms of propagating flexural wave and associated curvature waveforms of a pseudo-pristine beam-like structure can be estimated using polynomials that fit waveforms of the beam-like structure to be examined. A multi-resolution local-regression temporal-spatial curvature damage index is defined by comparing curvature waveforms of a damaged structure and those of a pseudo-pristine structure. High temporal-spatial curvature damage index values are expected in the neighborhood of damage and they can be inversely used to identify locality and extent of the damage. To further assist with the identification, a two-dimensional auxiliary temporal-spatial curvature damage index and a one-dimensional auxiliary curvature damage index are developed. A numerical investigation is conducted to study the proposed identification method. It is found that damage identification results depend on the size of an interval for fitting polynomials and there is an appropriately sized interval. Besides, the identification method is robust against measurement noise and errors and capable of indicating the locality and extent of damage in neighborhoods of high damage index values. The effectiveness of the proposed identification is validated in an experimental investigation. Future work could include a comparative study between the developed damage identification method and other existing methods with respect to their noise-robustness and damage-sensitivity.
